# Ocrelizumab in Multiple Sclerosis: A Real-World Study From Spain

**DOI:** 10.3389/fneur.2020.592304

**Published:** 2021-01-15

**Authors:** Angel P. Sempere, Leticia Berenguer-Ruiz, Ines Borrego-Soriano, Amparo Burgos-San Jose, Luis Concepcion-Aramendia, Lucian Volar, Miguel Aragones, Antonio Palazón-Bru

**Affiliations:** ^1^Neurology Service, Hospital General Universitario de Alicante, Instituto de Investigación Sanitaria y Biomédica de Alicante (ISABIAL), Alicante, Spain; ^2^Department of Clinical Medicine, Miguel Hernández University, San Juan de Alicante, Spain; ^3^Neurology Service, Hospital Marina Baixa, Villajoyosa, Spain; ^4^Pharmacy Department, Hospital General Universitario de Alicante, Instituto de Investigación Sanitaria y Biomédica de Alicante (ISABIAL), Alicante, Spain; ^5^Department of Radiology, Hospital General Universitario de Alicante, Alicante, Spain

**Keywords:** multiple sclerosis, drug therapy, ocrelizumab, safety, tolerability, real-world, effectiveness, MRI

## Abstract

**Objectives:** The aim of this study was to describe the tolerability, safety, and effectiveness of ocrelizumab for primary progressive multiple sclerosis (PPMS) and relapsing multiple sclerosis (RMS) in a clinical practice setting.

**Methods:** In this retrospective observational study, we analyzed clinical and MRI data in all patients with PPMS and RMS who had received at least one infusion of ocrelizumab in two health areas in south-eastern Spain. Patients involved in any ocrelizumab trial and those patients with a follow-up shorter than 6 months were excluded.

**Results:** The cohort included 70 patients (42 women) who had received ocrelizumab; 30% had PPMS and 70%, RMS. At baseline, patients' mean age was 47.1 years in the PPMS group and 39.2 years in the RMS group, while the median EDSS was 3.0 and 2.5, respectively. Median follow-up was 13.6 months. The median number of treatment cycles was three. Most patients remained free from clinical and MRI activity after ocrelizumab initiation. Baseline MRI showed T1 Gd-enhancing lesions in 57% of the patients; by the first MRI control at 4–6 months, all patients except one were free of T1 Gd-enhancing lesions (69/70, 98.6% *P* < 0.001). The proportion of patients with NEDA was 94% in the group of RMS patients who were followed for at least 1 year. Ocrelizumab was generally well-tolerated; the most common adverse events were infusion-related reactions and infections, none of which were serious.

**Conclusions:** Our real-world study supports the tolerability, safety, and effectiveness of ocrelizumab in clinical practice.

## Introduction

The humanized anti-CD20 B cell-depleting antibody ocrelizumab is approved in Europe for treating adults who have relapsing forms of multiple sclerosis (RMS) with active disease or early primary progressive multiple sclerosis (PPMS) with imaging features characteristic of inflammatory activity (1). The pool of PPMS patients who are candidates for this drug differs from the population studied in the pivotal phase 3 randomized clinical trials (RCT) with respect to the requirement of evidence of inflammatory activity from magnetic resonance imaging (MRI) (T1 Gd-enhancing lesions and/or new or enlarging T2 lesions), which was not present in the RCT inclusion criteria (2).

In the 96-weeks OPERA I and II trials in patients with RMS, ocrelizumab significantly reduced annualized relapse rates vs. interferon β-1a by 46% and the number of gadolinium-enhancing lesions by 94% (3). Likewise, in the ORATORIO trial in patients with PPMS, ocrelizumab significantly reduced the risk of confirmed disability progression relative to placebo (2). Ocrelizumab was generally well-tolerated in these studies, with mild to moderate infusion-related reactions and infections being the most common adverse events (4).

Although RCTs are essential to establish the efficacy of a new drug, they have limited validity because their results may not be widely generalizable, since the enrollment of patients with different comorbidities or previous treatments may be limited by the inclusion criteria. Real-world studies can thus provide useful information on the treatment tolerability, effectiveness and safety (5). Real-world data on ocrelizumab is limited as only a few studies have been published in Europe (6–8). The aim of this study was to describe the tolerability, safety and effectiveness of ocrelizumab for PPMS and RMS in clinical practice in a different geographical setting.

## Methods

### Patients and Study Design

This retrospective, observational study was performed in two health areas in the province of Alicante: Marina Baixa and Alicante, both situated in south-eastern Spain with a combined population of about 500,000. Patients with multiple sclerosis were attended at Marina Baixa General Hospital and Alicante General Hospital. The patients of both centers were evaluated jointly, under the same protocol. The healthcare system in Spain is universal and free at the point of service.

The main inclusion criteria was a history of initiation of ocrelizumab. Patients involved in any ocrelizumab trial and those patients with a follow-up shorter than 6 months were excluded. We retrospectively analyzed data in all patients with PPMS and RMS who had received at least one infusion of ocrelizumab. Multiple sclerosis was diagnosed according to the McDonald criteria (9). Clinical relapse, disease progression, and adverse events during ocrelizumab treatment were assessed by reviewing medical reports until September 18, 2020.

The standard patient follow-up included visits at 3, 6, and 12 months and every 6 months thereafter. During follow-up visits, clinicians considered new relapses and assessed patients using the Expanded Disability Status Scale (EDSS). Trained examiners with Neurostatus certification (APS, LBR) performed all EDSS assessments.

Patients underwent brain MRI scans before ocrelizumab initiation (baseline); at 4–6 months (before the second cycle of ocrelizumab), at 12 months, and at 24 months. Spinal cord and brain MRI scans, using 1.5 T and 3 T scanners, were done on an individual basis. At least contiguous, 3-mm axial sections, T2-weighted, FLAIR and gadolinium-enhanced T1-weighted scans through the whole brain were acquired in all patients according to published guidelines (10). MRI scans were read by experienced radiologists.

Baseline data collected from medical records were as follows: (a) demographic variables, (b) type of multiple sclerosis, (c) disease-modifying therapy before starting on ocrelizumab, (d) EDSS score, (e) number of relapses in the previous year, (f) time since diagnosis, (g) number of gadolinium-enhancing lesions on MRI, and (g) reason for starting ocrelizumab. Variables and outcomes assessed during follow-up were: (a) duration of follow-up, (b) number of relapses, (c) EDSS at the last visit, (d) number of ocrelizumab cycles, (e) adverse events, (f) number of gadolinium-enhancing lesions on the first MRI after ocrelizumab initiation (4–6 months), (g) number of new T2-lesions and T1 gadolinium-enhancing lesions in the annual MRI, and (h) discontinuation of ocrelizumab.

### Clinical and MRI Outcomes

A relapse was defined as new or recurrent symptoms and objective typical findings of multiple sclerosis with a duration of at least 24 h, in the absence of fever or infection (9). Disability progression was defined as a sustained (≥3 months) increase in the EDSS score, of: 1.5 points if the baseline EDSS score was 0; 1 point if the baseline score was 1–5.5; and 0.5 points if the baseline EDSS score was 6.0 or more. Disability improvement was defined as a sustained (≥3 months) decrease in the EDSS score, of: 0.5 points if the baseline EDSS score was 6.5 or more, or one point if the baseline score was 6.0 or less (11).

Clinical activity was defined as relapse and/or disability progression, and MRI activity was defined as the presence of T1 gadolinium-enhancing lesions at any time point or new T2 lesions on the annual MRI (compared to the MRI performed at 4–6 months). Highly active disease was defined as one or more relapse in the previous year and one or more T1 gadolinium-enhancing lesion on the baseline MRI.

No evidence of disease activity (NEDA) outcome was assessed in RMS patients who were followed for at least 1 year. NEDA status was defined as the combined absence of clinical (relapses and disability progression) and MRI activity (12).

### Treatment Protocol

Ocrelizumab was administered according to the schedule recommended in its summary of product characteristics (1). Before ocrelizumab administration, all patients were evaluated by their attending neurologist about symptoms suggestive of COVID-19 after Covid pandemic. The initial 600 mg cycle was administered as two separate intravenous infusions of 300 mg, at a 2-weeks interval. Subsequent cycles were administered as a single 600 mg intravenous infusion every 6 months. The premedication for all cases consisted of 100 mg intravenous methylprednisolone, 10 mg of cetirizine or 5 mg of dexchlorpheniramine, and 1,000 mg of paracetamol. Patients were monitored at hospital during the infusion and for 1 h after its completion. Infusion-related reactions included all symptoms and events occurring during or within 24 h of the infusion (in hospital or at home) and were graded as mild, moderate, severe, or life-threatening according to Common Terminology Criteria for Adverse Events (CTCAE) version 4.0 (13).

### Statistical Analysis

Quantitative variables are described using the mean ± standard deviation (SD) or median and range and they were compared with Student test or Mann–Whitney *U* depending on the normality of the distribution. Qualitative variables are presented as absolute and relative frequencies and were compared with chi-squared test. We compared the number of patients with T1 gadolinium-enhancing lesions on MRI at baseline and follow-up using McNemar's test. All calculations were performed with a statistical significance of 5% and for every relevant parameter, we calculated the confidence interval (CI) of 95%. The statistical package used was the IBM SPSS Statistics version 25.

## Results

### Cohort Characteristics

A total of 70 patients (42 female and 28 male), who had received at least the first cycle of ocrelizumab and with a follow-up longer than 6 months were included. There were no significant differences in baseline demographics and clinical characteristics (age, sex, EDSS, disease duration) for the two centers. Their clinical characteristics are summarized in Table 1. Twenty-one patients (30%) with a mean age 47.1 years had PPMS, and 49 patients (70%) with a mean age of 39.2 years, RMS. Relevant comorbidities according to the treating neurologist were present in 24% of patients (Table 2).

**Table 1:** Baseline characteristics in our cohort of 70 patients with multiple sclerosis treated with ocrelizumab.

**Patients**	**RMS (*n =* 49)**	**PPMS (*n =* 21)**
Age at ocrelizumab start	39.2 ± 10.9	47.1 ± 10.5
Sex (Female)	69%	38%
Time since diagnosis (years)	7.7 ± 6.7	2.8 ± 4.1
Baseline EDSS; median (IQR)	2.5 (2–3)	3.0 (3–4.8)
ARR previous year	1.3 ± 0.65	–
Treatment naive *n/N* (%)	10/49 (20%)	19/21 (90%)
Patients with at least one Gd-enhancing lesions, *n/N* (%)	31/49 (63%)	9/21 (43%)

*ARR, anualized relapse rate; EDSS, Expanded Disability Status Scale; IQR, interquartile range*.

**Table 2:** Comorbidities in patients treated with ocrelizumab (*n* = 70).

**Comorbidity**	***N* patients**
Bipolar disease	1
Cerebral palsy	1
Chronic migraine	1
Diabetes mellitus	2
Heart disease	2
Hepatitis B inactive carrier	1
Hodgkin's lymphoma in remission	1
Hypertension	2
Morbid obesity	2
Pituitary adenoma	1
Psoriasis	1
Thrombocythemia	1
Uveitis	1

The main reason for switching to ocrelizumab for RMS was treatment failure due to clinical relapse, MRI activity or both (36/39, 92%). One patient on fingolimod was switched due to hepatic toxicity, and another one (also on fingolimod) because of persistent vomiting after bariatric surgery. The patient on rituximab was switched due to serum sickness.

In the RMS group at baseline, median EDSS at was 2.5, the annualized relapse rate in the previous year was 1.3 ± 0.65, 63% (31/49) of patients had gadolinium-enhancing lesions on MRI, and 61% (30/49) had highly active disease. In the PPMS group at baseline, median EDSS was 3.0 and 43% (9/21) of the patients had gadolinium-enhancing lesions on MRI.

Ninety percent of PPMS patients were treatment-naive, compared to 20% of RMS patients. Before starting ocrelizumab, patients' most recent treatments included beta-interferon (*n* = 12), dimethyl fumarate (*n* = 11), fingolimod (*n* = 10), teriflunomide (*n* = 2), cladribine (*n* = 2), glatiramer acetate (*n* = 2), rituximab (*n* = 1), and alemtuzumab (*n* = 1). No patient switched from natalizumab to ocrelizumab. There was no washout period after beta-interferon and glatiramer acetate, but for patients on fingolimod, it was 1 month; on teriflunomide, 2 weeks, after undergoing the accelerated elimination procedure with cholestyramine; and on dimethyl fumarate, 1 week, except for one patient with lymphopenia that required a longer washout interval. The washout period for the patient on rituximab was 6 months, and the patient on alemtuzumab began ocrelizumab 16 months after the second cycle of alemtuzumab. No patient experienced a relapse during the washout period.

### Clinical Course After Treatment Initiation With Ocrelizumab

The clinical course was assessed in all the patients who began treatment with ocrelizumab, with a mean follow-up of 13.6 months (range 6–32). Follow-up was longer in the patients with PPMS compared to those with RMS (17 vs. 12 months, *p* < 0.05). No patient was lost to follow-up. The median number of treatment cycles was 3 (range 2–6).

The clinical and MRI outcomes after ocrelizumab initiation are outlined in Table 3. Among the 21 patients with PPMS, one patient (5%) experienced disability progression and discontinued treatment. In the 49 patients with RMS, only one had a relapse, none experienced disability progression, and nine showed disability improvement (18%, 95% CI 10–31%). The annualized relapse rate fell from 1.3 ± 0.65 before ocrelizumab initiation to 0.02 ± 014 after (*P* < 0.001). There was no evidence of clinical activity (relapses and/or disability progression) in 98% of RMS patients.

**Table 3:** Clinical and MRI outcomes (*n* = 70).

**Outcome**	
**Relapses in RMS patients**	
ARR 12 months prior to study inclusion	1.3
ARR after ocrelizumab initiation	0.02
*Disability progression (EDSS)*	1/70 (1.4%)
**MRI**	
Patients with Gadolinium-enhancing lesions at:	
Baseline	40/70 (57%)
4–6 months	1/70 (1.4%)
12 months	0/46 (0%)
New or enlarging T2-hyperintense lesions at 12 months	1/46 (2.2%)

*ARR, annualized relapse rate; RMS, relapsing multiple sclerosis*.

Baseline MRI showed T1 Gd-enhancing lesions in 57% of the patients (RMS: 63%, PPMS: 43%). All patients except one were free of T1 Gd-enhancing lesions at the first control MRI performed at 4–6 months (69/70, 98.6% *P* < 0.001). At the MRI at 12 months, all patients were free of T1 Gd-enhancing lesions (0/46, *P* < 0.001), and only one patient showed new T2 lesions compared to the previous MRI (2.2%).

The proportion of patients with NEDA was 94% (31/33) in the group of RMS patients who were followed for at least 1 year.

### Tolerance and Safety

Just over half (37/70, 53%) of the patients reported adverse events, none of which were serious (Table 4). The risk of adverse events was higher in the group of patients with previous DMT (59%) than in the group of patients who were treatment-naïve (45%) but the difference was not statistically significant (*P* = 0.257). The most frequent adverse events were infusion-related reactions: 43% (95% CI 32–55%) reported at least one; all of these were mild to moderate and were treated by reducing the infusion rate and administering symptomatic therapy if needed. The rate of this complication decreased from 40% (28/70) in the first cycle to 16% (11/70) thereafter. Aspirin 300 mg was included in the premedication protocol in some patients to prevent flushing.

**Table 4:** Adverse events in 70 patients treated with ocrelizumab.

**Adverse event**	***n* (%)**
Any adverse event	37 (53%)
Infusion-related reactions^*^	30 (43%)
Mild	14 (20%)
Moderate	16 (23%)
Severe	0
Infections	9 (13%)
Urinary tract infections	5
Pneumonia	1
Cellulitis	1
Gastroenteritis	1
Dental phlegmon	1
Others	2 (3%)
Alopecia areata	1
Biliary colic	1

* *Infusion-related reactions included pruritus, sore throat, rash, flushing, urticaria, erythema, headache, irritability and myalgias*.

Nine patients had infections: five had urinary tract infections and one each pneumonia, gastroenteritis, cellulitis, and dental phlegmon. No patient developed symptoms suggestive of COVID-19. No patient required hospitalization, and no malignancies were detected. The switch from rituximab to ocrelizumab due to rituximab-induced serum sickness was well-tolerated and the patient did not develop serum sickness after the first cycle (two infusions) of ocrelizumab.

Two patients (2.9%) discontinued ocrelizumab; one due to pregnancy and the other one because of lack of efficacy, but none did so because of an adverse event or tolerability.

## Discussion

Ocrelizumab has recently been approved in Europe for the treatment of patients with multiple sclerosis, but European data on its real-world use are limited (6–8). Our results support the safety and effectiveness of ocrelizumab in a clinical practice setting.

The results of clinical trials of ocrelizumab may not be generalizable to clinical practice if patients' baseline characteristics are significantly different from those of trial participants. With regard to age, disease duration and the percentage of treatment-naïve patients, our cohort of PPMS patients was similar to that in the ORATORIO phase 3 trial of ocrelizumab. The number of patients with gadolinium-enhancing lesions on the baseline MRI was slightly higher (43.5 vs. 27.5%). Only one of the 21 patients with PPMS in our cohort experienced confirmed disability progression (mean follow-up of 17 months). A recent real-world data study confirmed that ocrelizumab can stabilize disability progression in patients with PPMS and three out of 17 patients even showed clinically relevant improvement in disability status (8). In the ORATORIO trial, pre-specified non-powered subgroup analyses indicated that patients who were younger or had T1 Gd-enhancing lesions at baseline had a greater treatment benefit than older patients or those without T1 Gd-enhancing lesions, which may explain the low rate of disability progression in our cohort (14).

Our results confirm the rapid suppression of new focal brain MRI lesion activity with ocrelizumab. In our cohort, 98.6% of patients were free of T1 Gd-enhancing lesions at the first control MRI performed at 4–6 months. The analysis of phase 2 MRI data of the ocrelizumab 600 mg dose revealed near-complete suppression of T1 Gd-enhancing lesions by week 12 (15). MRI data were lacking in the already published ocrelizumab real-world studies (6–8).

The overall annualized relapse rate of patients with RMS in the study by Ellwardt et al. was 0.17 (95% CI 0.10–0.24), which was very similar to that of the OPERA 1 phase 3 clinical trial (0.16, 95% CI 0.12–0.20). In our cohort, the proportion of patients with NEDA was 94% in the group of RMS patients who were followed for at least 1 year. The greater treatment benefit observed in our study may be due to the higher number of patients with highly active disease (61%). Subgroup analyses comparing ocrelizumab and other disease-modifying therapies (natalizumab, alemtuzumab, fingolimod, cladribine, teriflunomide, and dimethyl fumarate) have found higher efficacy in patients with more active disease (16–22).

About three quarters of the RMS patients included in the OPERA trial were treatment-naive, and the most common previous therapies were interferon and glatiramer acetate (3). In contrast, in our cohort and in other observational studies most RMS patients had been previously treated with other disease-modifying therapies (6, 7). Nonetheless, prior treatment *per se* did not impact the magnitude of the beneficial effect of ocrelizumab although previous therapies in the pivotal trial and in the observational cohorts were rather different (16). Ocrelizumab in the observational cohorts showed efficacy not only after switching from first-line injectable treatments but also after switching from highly effective therapies such as alemtuzumab, natalizumab, fingolimod, and cladribine, although the participant numbers were small.

As observed in the phase 3 trials and in the real-world studies, mild to moderate infusion-related reactions and mild infections were the most common adverse events. The percentage of infusion-related reactions in our study (43%) was similar to that of the pivotal clinical trials (ORATORIO: 39.9%, OPERA 1: 30.7%, OPERA 2: 37.6%) and higher than in other observational studies (6, 7). The premedication protocol in the three observational cohorts included intravenous methylprednisolone, antipyretics, and antihistamines, but the dose of methylprednisolone was different: we used 100 mg as indicated in the summary of product characteristics while 250 mg was used in the other two studies. Whether the reason for the observed difference in the rate of infusion-related reactions resides in the different doses of methylprednisolone or underreporting from patients warrants further study.

The most common infections observed in the clinical trials of ocrelizumab were upper respiratory tract and urinary tract infections. Minor infections were reported in 8 and 5% of patients in other observational cohorts (6, 7). In our cohort, this proportion was higher (13%) which may be explained by the longer follow-up with ocrelizumab in our series. Besides, it is likely that in our case there was underreporting of upper respiratory tract infections since most patients do not consult their physicians for symptoms of nasopharyngitis. While most reported infections to date have been minor, there have been a few isolated case reports of severe viral infections, such as a fulminant hepatitis associated with echovirus 25 and HSV-2 encephalitis, in patients on ocrelizumab (23, 24). We did not observe any serious infections, while the rate was 1.3% in the OPERA trial and 6.2% in the ORATORIO trial (2, 3).

We observed a very low treatment discontinuation rate with ocrelizumab, consistent with findings from the phase 3 trials and other observational studies. Only two patients (2.9%) discontinued ocrelizumab; one due to pregnancy and the other one because of lack of efficacy, but none did so due to safety issues. The rate of treatment discontinuation due to adverse events was 3.2% in the 96-weeks OPERA 1 trial and 4.1% in the ≥120-weeks ORATORIO trial (2, 3). The annual discontinuation rate (3%) was lower for rituximab, another anti-CD20 B cell–depleting antibody, compared to other DMTs in patients with newly diagnosed RMS in a real-world study from Sweden (25).

The effectiveness of ocrelizumab in this study is similar to that of rituximab in a similar general hospital setting although there are some differences concerning secondary infectious adverse events and discontinuation rate (26). The discontinuation rate in this study was 2.8% and no patient required hospitalization due to infectious adverse events while the discontinuation rate was 14.4% in the rituximab observational study from Sweden and four patients (4.8%) required hospitalization due to infectious adverse events. However, the follow-up was longer and the patients slightly older in the Swedish cohort which may explain the observed differences.

The main limitations of this study are the small sample, its retrospective design, a short time of follow-up and the absence of a control group. On the other hand, the study provides MRI and NEDA data that are not available from other real-world studies and a longer time of follow-up. Besides, the study was conducted in a general hospital setting with universal healthcare access, eliminating the bias of a tertiary referral center or unequal access to healthcare or DMTs.

In conclusion, our data confirm the short-term effectiveness, tolerability, and safety of ocrelizumab in real-world clinical practice. Further studies are needed to assess patient outcomes with longer follow-up periods.

## Data Availability Statement

The raw data supporting the conclusions of this article will be made available by the authors, without undue reservation.

## Ethics Statement

The studies involving human participants were reviewed and approved by the Institutional Ethics Committee of the Hospital General Universitario de Alicante (reference number: PI-2019-116). The patients/participants provided their written informed consent to participate in this study.

## Author Contributions

AS and LB-R: study design, analysis, drafting and revision of manuscript. IB-S: study design and drafting. AB-SJ: revision of manuscript. LC-A and LV: MRI analyses and revision of manuscript. MA: contributed patients and revised the manuscript. AP-B: performed the statistics and revised the manuscript. All authors contributed to the article and approved the submitted version.

## Conflict of Interest

LB-R has received personal compensation for consulting, serving on a scientific advisory board or speaking with Almirall, Biogen Idec, Merck Serono, Novartis, Roche, Sanofi-Aventis, and Teva. AS has received personal compensation for consulting, serving on a scientific advisory board or speaking with Almirall, Biogen Idec, Bayer Schering Pharma, Merck Serono, Novartis, Roche, Sanofi-Aventis, and Teva. The remaining authors declare that the research was conducted in the absence of any commercial or financial relationships that could be construed as a potential conflict of interest.
